# Comparative Study of Ultrasonication-Induced and Naturally Self-Assembled Silk Fibroin-Wool Keratin Hydrogel Biomaterials

**DOI:** 10.3390/ijms17091497

**Published:** 2016-09-07

**Authors:** Trang Vu, Ye Xue, Trinh Vuong, Matthew Erbe, Christopher Bennet, Ben Palazzo, Lucas Popielski, Nelson Rodriguez, Xiao Hu

**Affiliations:** 1 Department of Physics and Astronomy, Rowan University, Glassboro, NJ 08028, USA; vup77@students.rowan.edu (T.V.); vuongt27@students.rowan.edu (T.V.); erbem43@students.rowan.edu (M.E.); bennettc0@rowan.edu (C.B.); palazz63@students.rowan.edu (B.P.); popiel31@students.rowan.edu (L.P.); rodrig41@students.rowan.edu (N.R.); 2 Department of Biomedical Engineering, Rowan University, Glassboro, NJ 08028, USA; xuey5@rowan.edu; 3 Department of Chemical Engineering, Rowan University, Glassboro, NJ 08028, USA; 4 Department of Biomedical and Translational Sciences, Rowan University, Glassboro, NJ 08028, USA

**Keywords:** silk fibroin, wool keratin, hydrogel, DSC, FTIR, SEM, AFM

## Abstract

This study reports the formation of biocompatible hydrogels using protein polymers from natural silk cocoon fibroins and sheep wool keratins. Silk fibroin protein contains β-sheet secondary structures, allowing for the formation of physical cross-linkers in the hydrogels. Comparative studies were performed on two groups of samples. In the first group, ultrasonication was used to induce a quick gelation of a protein aqueous solution, enhancing the ability of *Bombyx mori* silk fibroin chains to quickly entrap the wool keratin protein molecules homogenously. In the second group, silk/keratin mixtures were left at room temperature for days, resulting in naturally-assembled gelled solutions. It was found that silk/wool blended solutions can form hydrogels at different mixing ratios, with perfectly interconnected gel structure when the wool content was less than 30 weight percent (*wt* %) for the first group (ultrasonication), and 10 *wt* % for the second group (natural gel). Differential scanning calorimetry (DSC) and temperature modulated DSC (TMDSC) were used to confirm that the fibroin/keratin hydrogel system was well-blended without phase separation. Fourier transform infrared spectroscopy (FTIR) was used to investigate the secondary structures of blended protein gels. It was found that intermolecular β-sheet contents significantly increase as the system contains more silk for both groups of samples, resulting in stable crystalline cross-linkers in the blended hydrogel structures. Scanning electron microscopy (SEM) and atomic force microscopy (AFM) were used to analyze the samples’ characteristic morphology on both micro- and nanoscales, which showed that ultrasonic waves can significantly enhance the cross-linker formation and avoid phase separation between silk and keratin molecules in the blended systems. With the ability to form cross-linkages non-chemically, these silk/wool hydrogels may be economically useful for various biomedical applications, thanks to the good biocompatibility of protein molecules and the various characteristics of hydrogel systems.

## 1. Introduction

A hydrogel is a three-dimensional, cross-linked polymeric network extensively swollen in water. Hydrogels can retain a significant amount of water molecules within their structure without being dissolved, thanks to their special molecular network [1]. The hydrophilic functional groups (such as –OH, –CONH, –CONH_2_, and –SO_3_H) attached to the hydrogel’s polymeric backbone give it the ability to absorb water, while the cross-linkers between its network chains help in resisting dissolution [1,2]. The cross-linkers in hydrogel structures belong to two categories: physical (entanglements or crystallites) or chemical (tie-points and junctions). These cross-linkers are provided by covalent bonds, hydrogen binding, van der Waals forces, or physical entanglement [2].

Hydrogels can be classified in numerous ways, based on their raw material sources, polymeric composition, physical structure, cross-linking, chemical composition, physical appearance, type of electrical charge network, or method of creation [1,2]. Traditional hydrogels have been widely used in drug releasing or in scaffolding for tissue engineering [3]. Recently, protein-based hydrogels have drawn increased attention, due to their unique structural and mechanical characteristics, which are similar to those of human extracellular matrices (ECM) [1,2,3,4]. There are various ways to create physical and chemical cross-linkers for protein hydrogels [1,2,3,4]. In this paper, the two techniques used to create hydrogels are ultrasonication and natural self-assembly. These rapid and economical physical methods accelerate the solution-gelation (sol-gel) transition of a protein by inducing physical cross-linker formation in the gel network. Both methods, which can be controlled without the addition of chemical processes, are effective and efficient for medical applications [4,5].

Among various biomaterials for hydrogels, silk is a natural fibrous protein that has been widely used for decades in textile materials, biomedical sutures, cosmetics, and food additives [6]. In recent years, silk proteins have been applied to bioelectronics, to the fabrication of 3D porous scaffolds for bone tissue reconstruction, and to 3D matrices for in vivo tumor models [7,8,9]. Silks can be produced by many different insects. One of the most studied silks comes from the *Bombyx mori* silkworm, which contains two main proteins, sericin (20–30 *wt* %) and fibroin (70–80 *wt* %), in addition to other waxes, carbohydrates, and inorganic impurities [10]. Silk fibroin is a long, fibrous protein composed of repeating motifs of Gly-Ser-Gly-Ala-Gly-Ala. In its secondary structure, silk fibroin molecules are organized in layers of antiparallel β-sheets held together by intermolecular hydrogen bonds. Each fibroin fiber is coated by sericin, which acts as a glue, holding the structure of the fiber network together [4]. There are three types of silk structure, classified as either Silk I (random coils), Silk II (organized β-sheets, insoluble in water), or Silk III (formed at solution-air interface). These structures aid in the fabrication of three-dimensional silk materials, which in turn have the capability of shaping specific tissue ECM structures in the body [11,12,13].

Wool keratin materials are also significantly useful for hydrogel applications. Keratin is a large group of homologous proteins that have filamentous structure. They have two types of protein sheet arrangement: α-keratin and β-keratin. While α-keratin is found in hair, claws, and wool, β-keratin, being stiffer, is usually found in claws, shells, and horns [14]. Keratin exhibits different organizational structures: monomer, heterodimer, tetramer, octamer, and intermediate filaments. The first level is a protein secondary structure of Keratin I and II monomers, which are held together by hydrogen bonds and form a left-handed α-helix. A heterodimer, the second level of the structure, is a right-handed coiled-coil between one monomer of each type twisted around each other. Two heterodimers can associate with each other to form a tetramer; two tetramers together can form an octamer; and so on. The final intermediate filaments contain a central domain with four segments in α-helical conformations, which are separated by three short-link segments predicted to haveβ-turn configuration. The filaments consist of multiple keratin monomers, held together by hydrogen bonds, hydrophobic interactions, and disulfide bridges. The fibrous keratin also has the ability to form cysteine, which is insoluble to many acids as well as immune to proteolytic enzymes.

Both silk fibroin and keratin are mechanically stable, biocompatible, and biodegradable. They are capable of self-assembly and forming hydrogels with unique characteristics. Silk fibroin has resilience, flexibility, and excellent mechanical strength, while keratin is mechanically inferior, rigid, and degrades quicker than fibroin. Silk fibroin has been used as a substitute for anterior cruciate ligaments, or as a suture for biocompatible gauze, allowing cell growth in skin burn cases [15,16]. However, keratin shows far superior biological activities in cellular recognition and behavior, such as cell encapsulation and bioprinting [17,18]. Keratin is able to self-assemble into three-dimensional structures, rapidly enhancing cellular adhesion and proliferation [15,19]. Since silk fibroin has the ability to form β-sheet crystal networks during the gelation process, it can work as a physical cross-linker, connecting other non-cross-linked biomaterials such as keratin. This gives the blended fibroin/keratin both mechanical strength and excellent biological activities [4,15,20]. Therefore, when blended together to form a hydrogel, the fibroin/keratin mixture exhibits the properties of the individual components, as well as additional properties resulting from the unique intermolecular interactions between fibroin and keratin.

Both fibroin and keratin have shown a wide range of uses in biomedical applications. Thus, the development of a hydrogel biomaterial, coming from a silk/keratin blend, will greatly benefit regenerative medicine and tissue engineering. This composite can be utilized as a scaffolding material for bone and cartilage regeneration, as a support for cell adhesion and growth in vivo, and as a dermal substitute for skin tissue engineering [15,17,21]. It was suggested that physically cross-linked hydrogels, as in fibroin/keratin, are more attractive for entrapping sensitive molecules (such as cells or cytokines), because toxic reactive molecules are avoided in the cross-linking process, as opposed to chemically cross-linked hydrogels [4,22,23,24,25]. In addition, silk/keratin hydrogels can aid in the regeneration of nerve cells and soft tissue, and can also act as a hemostat to stop bleeding in liver lacerations [4,26]. Moreover, silk/keratin hydrogel blends can be used as a rate-controlled drug delivery system. Therefore, combining silk fibroin and wool keratin together in a hydrogel format may result in a tunable biomaterial with properties and applications that cannot be achieved using currently available polymer/protein materials.

## 2. Results and Discussion

### 2.1. Structural Analysis

**Figure 1:**
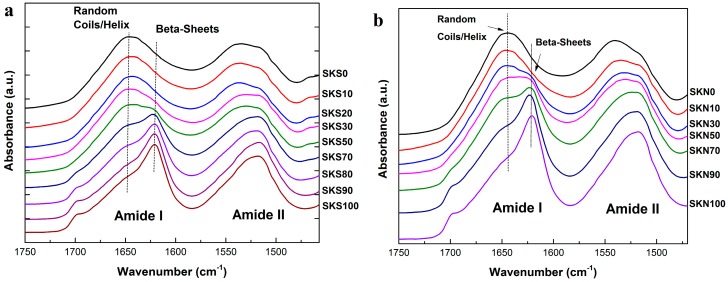
FTIR absorbance spectra of various silk-keratin samples: (**a**) ultrasonication-induced hydrogel/aggregate systems (SKS0, SKS10, SKS30, SKS50, SKS70, SKS80, SKS90, SKS100); (**b**) naturally self-assembled hydrogel/aggregate systems (SKN0, SKN10, SKN30, SKN50, SKN70, SKN90, SKN100).

FTIR was first performed on both groups of samples to study the structures of the hydrogel systems, which contain different compositions of silk and wool at the molecular level. Figure 1a,b shows the FTIR absorbance of ultrasonicated samples (SKS0, SKS10, SKS20, SKS30, SKS50, SKS70, SKS80, SKS90, and SKS100) and naturally assembled samples (SKN0, SKN10, SKN30, SKN50, SKN70, SKN90, and SKN100), respectively, for the wavenumber region of 1500–1750 cm^−1^ (characteristic peaks of amide backbones).

Generally, the intense band at 1500–1700 cm^−1^ was assigned to the peptide backbone of amide I (1700–1600 cm^−1^) and amide II (1600–1500 cm^−1^) absorptions. Since the amide I region is mainly the stretching effects of C=O vibrations (>80%) [4,27,28,29], it can be directly used to determine the protein secondary structures quantitatively. Spectrum bands in the amide II region mostly come from the out-of-phase combination of CN stretching and NH bending vibrations. The characteristic Amide I peak contains different secondary structures, including: strong intermolecular β-sheet (1622–1627 cm^−1^), strong intramolecular β-sheet (1628–1637 cm^−1^), weak β-sheet (1696–1703 cm^−1^), random coils (1637–1644 cm^−1^), alpha helix (1645–1654 cm^−1^), and β-turns (1655–1695 cm^−1^) [15]. In a mixed solution of fibroin and keratin, β-sheet crystal is a critical key factor in the sample gelation process, since these β-sheets can form intermolecular hydrogen bonds to entrap and capture silk and keratin filaments. When there are enough intermolecular hydrogen bonds, the solution will form a hydrogel network using β-sheet crystals as the physical cross-linkers [4,27,28,29].

For the spectra of silk/keratin hydrogels from both groups (ultrasonication and natural hydrogels, as shown in Figure 1a,b, respectively), with the increase of silk fibroin content in the blend solution, the characteristic peaks of the β-sheet (around 1625 cm^−1^) in the amide I region increased significantly [27,28,29]. Since wool keratin could not form a large amount of strong β-sheet crystals, the β-sheet formed here can be set as an indicator for the silk fibroin crystalline structure. In the amide I region, the main peak position shifted from 1640 to 1625 cm^−1^ (random coils to intermolecular β-sheet crystals) with the increase of silk content, indicating that increasing the silk fibroin concentration in the mixture results in an increased structural insolubility and stability in the blend. Generally, with the increase in silk content, the 1518 cm^−1^ peak in the amide II region also increased significantly, mostly contributed from the tyrosine side chain vibrations in the protein network [27,28,29].

FTIR results also indicated that both groups of samples show strong intermolecular β-sheet peaks when the silk concentration is 70% or higher. As mentioned previously, β-sheet crystalline contents in the protein blends play a key role in the formation of hydrogel networks. Therefore, for both groups of samples, the percentage of the secondary structure in each fibroin/keratin system was obtained by a FSD curve fitting method [4], as shown in Table 1 and Table 2 In the pure silk hydrogel scaffolds, 54.5% and 52.4% of β-sheet crystals are presented in the ultrasonication-induced and natural self-assembly samples, respectively, which indicates that both gelation methods need a similar amount of crystals to form stable cross-linkers for the pure silk fibroin hydrogel chains. However, in both groups of samples, as the concentration of keratin increases (silk fibroin composition decreases), the percentage of β-sheets decreases gradually. The lowest β-sheet fractions present in the two groups are 25.5% and 22.6% within pure keratin samples, SKS0 and SKN0, respectively. Since both silk fibroin and wool keratin may contain a small amount of β-sheets in their regular structure, it is not possible to calculate the exact amount of β-sheet crystals in the cross-linkers. However, data from Table 1 and Table 2 suggest that the overall percentage of β-sheet cross-links increases in the blend system with the increase of fibroin composition for both methods. Since a higher ratio of silk in the sample allows fibroin chains to stay closer to each other in space, the formation of cross-linkers using β-sheet crystals would become easier in the solution.

### 2.2. Morphology Analysis

**Figure 2:**
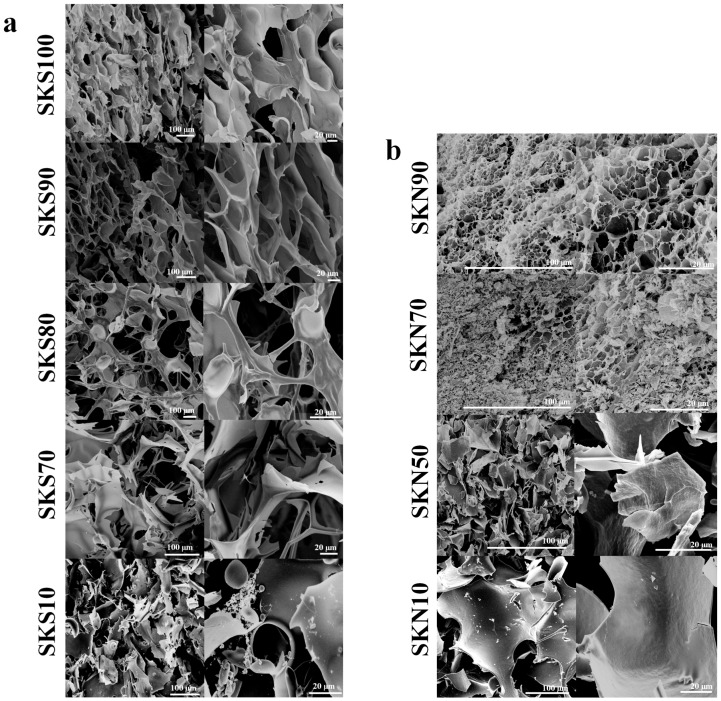
SEM images of various silk-keratin samples: (**a**) ultrasonication-induced hydrogel/aggregate scaffolds (with various silk fibroin composition of 100, 90, 80, 70, and 10 *wt* %); (**b**) naturally-self-assembled hydrogel/aggregate scaffolds (with various silk fibroin composition of 90, 70, 50, and 10 *wt* %). All samples are displayed at two scales: 100 and 20 μm.

SEM was used to examine the morphologies of the silk/wool hydrogel scaffold samples from both groups, ultrasonication-induced and naturally self-assembled. As mentioned in the previous section, the pure silk fibroin has a fibrous morphology, containing antiparallel β-sheets, as shown in Figure 2a (SKS100). In the fibroin/keratin hydrogel composite, created using ultrasonication, these β-sheets act as cross-linkers to create a continuous network from the randomly oriented silk and keratin fibers. In Figure 2a, systems containing at least 70 *wt* % silk appear to have areas of regular, three-dimensional, interconnected structure. SKS100, SKS90, SKS80, and SKS70 hydrogel scaffolds have an average pore size of 20–50 μm. As the relative amount of silk fibroin was reduced, so was the relative amount of β-sheet cross-linkers, resulting in fewer interconnections. Below 70 *wt* % of silk, the formed hydrogels/aggregates tend to be structurally unstable. Thus, they would be easily broken under a mechanical press. At 10 *wt* % silk content (SKS10), the flake surface of keratin was smooth with small holes but had no regular interconnected gel structure (Figure 2a). Many small spherical structures were also seen in this sample, which could be micelle structures from silk proteins [30].

In the second group, samples were slowly gelled at room temperature. Only systems with at least 90 *wt* % silk exhibited a homogenous and continuous porous structure with a large number of cross-linked sheets, as shown in Figure 2b. SKN90 morphology has an average pore size of 2–5 μm, which is 10 times smaller than the average pore size of hydrogel samples generated by ultrasonic waves. This could be due to differences in the gelation mechanisms between the two groups. In the ultrasonication method, fibroin and keratin chains interacted with each other immediately without phase separation in the aqueous solution, and then were quickly gelled (cross-linked) under sonication. While in the natural self-assembly method, it is believed that silk fibroin and keratin chains were first phase separated in the nanoscale and formed dense aggregates in the aqueous solution over the two days, then they slowly interacted with each other to form gel cross-linkers in the microscale. Because they are more tightly packed together and closer in space, the pore size of the self-assembled systems is much smaller than the ultrasonication ones. In Figure 2b, systems with less than 90 *wt* % silk show a non-homogeneous, flake-like morphology. For example, SKN70 morphology displays both porous and flake-like regions, suggesting imperfect hydrogel networks. Close observation of Figure 2b also shows that the flake size increases as the silk concentration decreases in the network, with an average length of 250 µm for the 10 *wt* % silk sample (SKN10).

The FTIR results in Table 1 suggest that a total β-sheet crystallinity of at least 45% is required in both ultrasonication-induced and naturally self-assembled samples to form a mechanically stable fibroin/keratin hydrogel system (e.g., SKS100, SKS90, SKS80, and SKN90).

**Figure 3:**
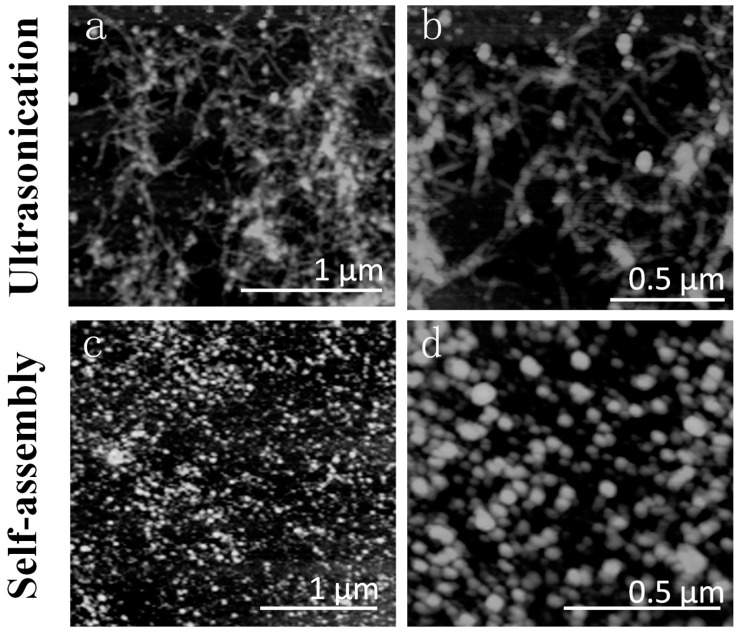
AFM images of SKS50 (**a**,**b**) and SKN50 (**c**,**d**) samples with the same silk composition (diluted to 0.01 *wt* %). Both samples are shown at two scale bars: 1 and 0.5 µm.

To further understand the morphology of the fibroin/keratin blends at the nanoscale, AFM was used to study particle-particle interactions between fibroin and keratin chains. Two samples with the same 50 *wt* % silk concentration, from both groups (ultrasonication-induced (SKS50) and naturally self-assembled (SKN50)), were diluted to 0.01 *wt* % and cast on a flat mica surface for comparison. Results are presented in Figure 3 with two scale bars, 1 µm and 0.5 µm, respectively. The silk concentration of both samples was lower than the minimum silk concentration required for the fibroin/keratin system to form a completely cross-linked insoluble hydrogel network (70 *wt* % for the first group and 90 *wt* % for the second group). In this way, the aggregates can be diluted to a smaller concentration for us to study the fibroin/keratin particle–particle interactions in the nanoscale, in the absence of hydrogel cross-linkers. As can be seen in Figure 3, light dots in the AFM images are due to fibroin/keratin particles, which have a typical size of 50–100 nm. With the aid of ultrasonication (Figure 3a,b), these particles interact with each other, forming interconnected networks at the nanoscale. These networks are fibroin/keratin blended chains, which may contain β-sheet structures. These blend chains are symbolized by green curves (as shown in the third column). On the other hand, without the aid of ultrasonication (Figure 3c,d), AFM images of the self-assembled sample (SKN50) show no particle–particle interactions. Thus, it can be interpreted that in natural self-assembled systems, fibroin and keratin chains do not strongly interact with each other, and are not blended very well in the nanoscale. Further investigation of sample pairs with other silk concentrations (25 *wt* %, 10 *wt* %) also shows similar results, indicating that the sonication method will not only create hydrogel cross-linkers that maintain the mechanical stability of the gel structure in the microscale, but also forces the silk and keratin molecular chains to interact with each other and form stable interconnected structures at the nanoscale.

### 2.3. Thermal Analysis

DSC and temperature-modulated DSC (TM-DSC) were performed to gain a better understanding of the miscibility of the ultrasonication-induced fibroin/keratin blend gel scaffolds at different mixing ratios. DSC is one of the most commonly used techniques for studying blend miscibility and thermal properties of polymer composites. The glass transition temperature of a system of two fully miscible polymers can be predicted using the Flory–Fox equation, shown in Equation (1):

(1) $$\frac{1}{T_{g}}=\frac{W_{1}}{T_{g1}}+\frac{W_{2}}{T_{g2}}$$

According to the Flory–Fox equation (Equation (1)), a fully miscible polymer blend exhibits a homogeneous amorphous phase at the micro-/nanoscale, where a single glass transition temperature (*T_g_*) can be predicted for this blend [31,32]. The glass transition temperature of the blend can either be between or beyond the glass transition temperatures of the two pure polymers [31].

**Figure 4:**
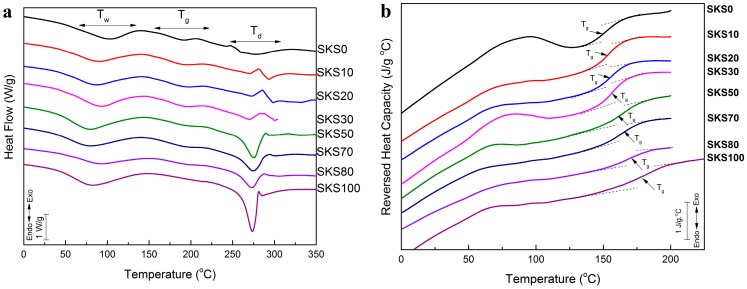
(**a**) Standard DSC scans of ultrasonicated silk/keratin hydrogel/aggregate scaffold samples (SKS10, SKS20, SKS30, SKS50, SKS70, and SKS80) and controls (pure wool (SKS0) and pure silk (SKS100)). The samples were heated at 2 °C·min^−1^ from 30 to 350 °C; (**b**) Reversing heat capacities of the samples from 0 to 200 °C (250 °C for pure silk), measured by TM-DSC with a 2 °C·min^−1^ heating rate, a modulation period of 60 s, and a temperature amplitude of 0.318 °C.

Figure 4a shows the standard DSC scans of the ultrasonication-induced fibroin/keratin systems (SKS100, SKS80, SKS70, SKS50, SKS30, SKS20, SKS10, and SKS0). All of these samples showed water peaks around 80 °C, similar to silk-bound water films studied previously [28,29]. No separated individual glass transition or degradation region for the keratin or silk components was observed in the blends, indicating that silk fibroin and keratin chains were well blended in a homogeneous system without phase separation. A degradation peak between 273 and 300 °C was observed for all samples (Table 3). While pure keratin and pure fibroin samples showed degradation peaks at 274.3 and 273.1 °C, respectively, all other samples showed higher degradation peaks (274–300 °C). The higher degradation peaks may suggest that ultrasonication-induced fibroin/keratin blends are more thermally stable than pure silk or keratin polymers, due to the interconnected structure formed at the nanoscale.

In TMDSC, the “reversing heat capacity”, which represents the reversible heat effect within the temperature range of the modulation, can be measured. The modulation of *T_S_*(*t*) with amplitude *A_Ts_* and period *p* (*ω* = 2π/*p*) can be obtained using Equation (2):

(2) $$T_{s}\left(t\right)=T_{0\;}+<q>\left[C_{s}/K\right]+A_{Ts}sin\left(\omega t-\varepsilon \right)$$

where *ɛ* is the phase shift related to the internal reference frequency, *K* is the Newton’s law constant, *C_S_* is the heat capacity of the sample calorimeter, <*q*> is the underlying heating rate, and *T*_0_ is the starting temperature. So the apparent reversing heat capacity *C_P_* can be expressed by Equations (3) and (4):

(3) $$C_{P}=[<A_{ɸ}>/<A_{Ts}>\omega ]K\left(\omega \right)$$

(4) $$K\left(\omega \right)=\sqrt{1+\tau^{2}\omega^{2}}$$

where <*A*_ɸ_> is the amplitude of the heat flow rate in a modulation cycle; *<A_Ts_>* is the modulation amplitude of the temperature (*T_S_*) with the frequency *ω*; and *K*(*ω*) is a calibration factor, with *τ* being a correction value used at the given conditions of the measurement (Equation (4)), which was determined from the sapphire calibration scans.

Figure 4b shows the reversing heat capacity curves of fibroin/keratin blends measured by TM-DSC. The glass transition temperature (*T_g_*) increased as the concentration of silk fibroin in the sample increased (Table 3), with the lowest sample’s glass transition temperature at 153.5 °C (pure wool keratin), and the highest at 183.9 °C (pure silk fibroin). With the increase of silk content from 0 to 100 *wt* %, the heat capacity increments at *T_g_* (∆*C_p_*) of fibroin/keratin blends also increased gradually from 0.159 to 0.250 J·g^−1^·°C^−1^ (Table 3).

**Figure 5:**
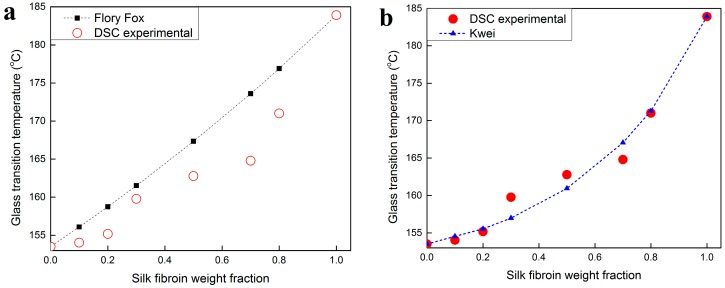
(**a**) Comparison between theoretical glass transition temperatures calculated using the Flory–Fox equation and the experimental glass transition temperatures collected using TM-DSC results; (**b**) comparison between theoretical glass transition temperatures calculated using Kwei equation (with *k* = 2.6 and *q* = −4) and the experimental glass transition temperatures collected using TM-DSC results.

Different classical equations, such as the Flory–Fox equation (Equation (1)) and the Kwei equation (Equation (5)), can be used to theoretically predict the glass transition temperature of polymer blends at different mixing ratios. The Kwei equation (Equation (5)) is a modification of the Flory–Fox equation:

(5) $$T_{g}=\frac{W_{1}T_{g1}\;kW_{2}T_{g2}}{W_{1}\;+kW_{2}}+qW_{1}W_{2}$$

in which *T_g_* is the glass transition temperature of the final polymer blend; *T_g_*_1_ and *T_g_*_2_ are the glass transition temperatures of the individual components, respectively; *W*_1_ and *W*_2_ are the weight fractions of these two components; *k* is the molecular interaction strength parameter; and *q* is the mixture specific intermolecular interaction (such as H-bonding) parameter. Subscripts 1 and 2 stand for pure silk fibroin (SKS 100) and pure keratin (SKS0) in this study, respectively. The values of *q* can be either positive or negative [33]. Figure 5a,b show the glass transition temperatures obtained by the three methods: experimental DSC, Flory–Fox, and Kwei. When using the Kwei equation, the closest parameters that fit the DSC experimental data are *k* = 2.6 and *q* = −4, resulting in theoretical *T_g_s* values that fit the experimental *T_g_s* very well. The parameter *k* represents the unequal contribution of each component in the mixture to the final *T_g_*. Parameter *k* has a valid physical meaning only when the mixture has ideal volume mixing and no interaction between components. Therefore, in real (non-ideal) mixtures, with varying degrees of intermolecular interaction, *k* value is mainly used for data fitting only [33]. The parameter *q* is a constant that represents the intermolecular interactions between the two components of the polymer mixtures. If *q* > 0, the two components should have strong attractive force between them [34]. In our case, a negative *q* means that fibroin molecules would not attract keratin molecules naturally, meaning there are no strong inter-associations such as H-bonds between the two types of molecules. Using ultrasonication, we are able to obtain a fully miscible (single *T_g_*) blended system because the ultrasonic waves push the silk and keratin molecules together in a short time. Using the naturally self-assembled process, it is expected that the fibroin and keratin chains will phase separate in the solution after several days.

### 2.4. Gelation Mechanism and Condition

**Figure 6:**
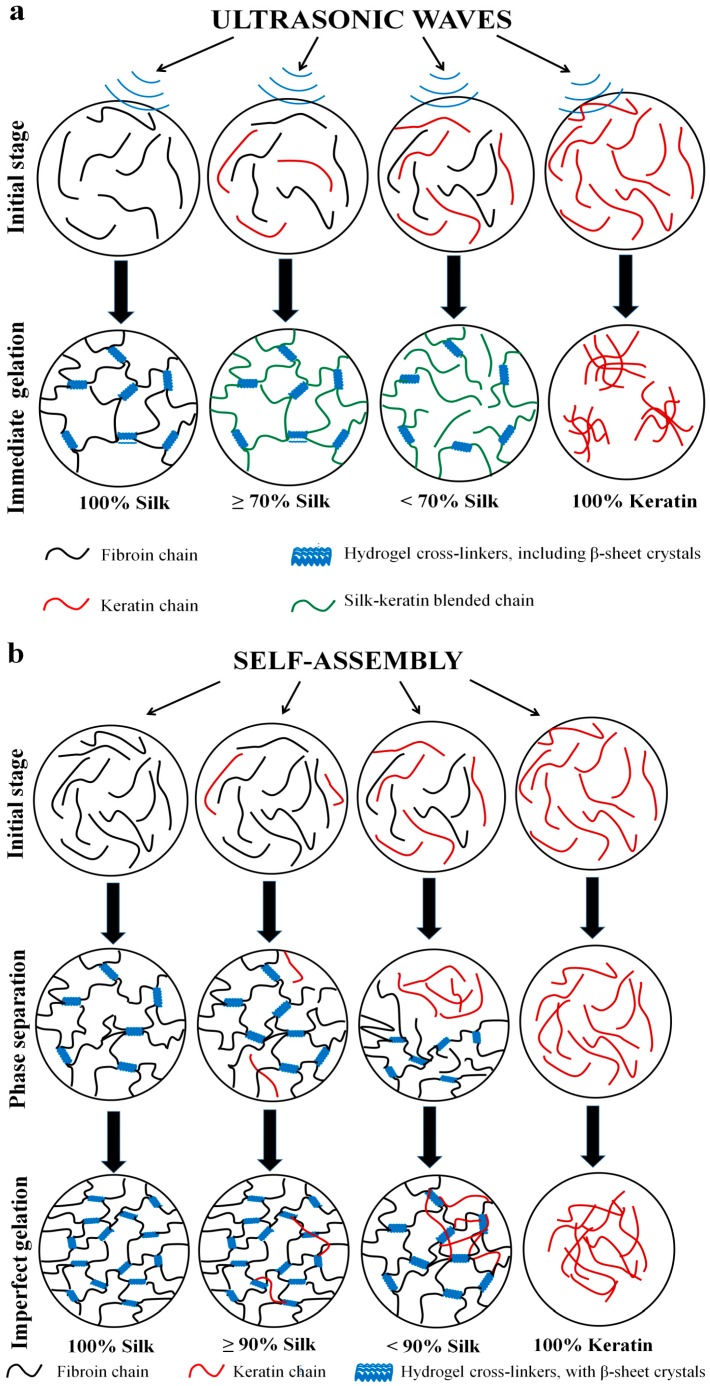
Hydrogel formation and structural mechanism for: (**a**) Ultrasonication-induced silk/keratin hydrogels. Row 1: Initial blends of silk fibroin and wool keratin during sonication, with pure silk and pure wool solution as controls; Row 2: After quick sonication, β-sheet crystals form cross-linkers immediately, entrapping keratin and silk chains at both the nano- and microscale. The blends have a homogeneous silk/wool gel structure when there is at least 70 *wt* % of silk (30 *wt* % of wool keratin). Below 70 *wt* % of silk, homogenous blend solutions cannot form continuous gel structures due to a lack of cross-linkers; (**b**) Naturally self-assembled silk/wool hydrogels. Row 1: Initial blend solutions of silk fibroin and wool keratin, with pure silk and pure wool solution as controls; Row 2: after a long suspension, macrophase separation of protein chains may occur at the nanoscale, with silk chains starting to form cross-linkers through self-assembled beta-sheets crystals; Row 3: After 12 h, solutions with less than 90 *wt* % silk (10 *wt* % wool or less) started to exhibit an imperfect gelation network.

In order to fully understand the gelation mechanism of the fibroin/keratin blends, results are analyzed at both the nanoscale and the microscale. At the nanoscale, it is important to study the particle–particle interactions that lead to a well-blended, homogeneous composite of fibroin and keratin chains (Figure 3). On the other hand, microscale study allows us to understand the role of β-sheets as physical cross-linkers, entrapping fibroin and keratin chains together to form a hydrogel network (Figure 1 and Figure 2). Systems with enough β-sheet cross-linkers to form network (microscale) and well-blended fibroin-keratin chains (nanoscale) result in stable and homogenous hydrogels. The mechanisms of silk-keratin gelation using the two methods are proposed in Figure 6a,b.

#### 2.4.1. Nanoscale Level

As we have seen in AFM images (Figure 3), with the aid of ultrasonication, fibroin and keratin particles can connect to each other to become filament networks. DSC results are consistent with AFM morphological observations, indicating that the fibroin and keratin molecular chains are fully miscible without phase separation for the ultrasonication-induced gel scaffolds. Therefore, we can symbolize the miscible fibroin-keratin molecules as green curves in Figure 3 and Figure 6a. On the other hand, without the aid of ultrasonication, naturally self-assembled samples show no fibroin/keratin particle–particle interconnections (Figure 3). Thus, phase separation in the blend may occur between the silk and keratin molecules in the early stage before the completion of gelation/aggregation after several days. We symbolize these individual fibroin and keratin molecules as black (silk fibroin) and red (wool keratin) curves in Figure 3 and Figure 6b. These early-stage aggregations make the pore size of the final hydrogels smaller with inhomogeneous distributions (Figure 6b).

#### 2.4.2. Microscale Level

At the microscale, for both methods, the mechanism of gelation in silk fibroin/keratin hydrogels can be explained by the formation of β-sheet crystals as physical cross-linkers. Wool keratin is an intermediate filament that cannot form physical cross-links during sonication, thus it cannot undergo gelation under different conditions (Figure 6a,b). Therefore, the formation of the hydrogel was controlled by the silk fibroin’s ability to create physical cross-links. Ultrasonication gives the samples energy, causing the silk and keratin chains to move around and become entangled with each other, forming cross-linked β-sheet crystals (Figure 6a). A silk fibroin composition of at least 70 *wt* % is necessary to provide enough β-sheet crystals (43%–45% in the blend, see Table 1) to achieve a fully connected, stable hydrogel system (Figure 6a,b). When the fibroin weight percent is less than 70%, there are not enough β-sheet crystals to effectively link all of the fibroin-keratin chains together (Figure 6a,b).

In the natural self-assembly process, many keratin chains may be partially phase separated from fibroin chains. The silk molecules will be closer to each other, allowing the silk fibroin to form β-sheet cross-links without the aid of ultrasonic waves. However, as previously shown in the AFM results, there were no particle–particle interactions in the naturally self-assembled gel system, meaning that fibroin and keratin chains could remain unblended or partially blended in the hydrogel (Figure 6b). This phase separation happened before the fibroin started to form cross-linkers with each other. Keratin chains, being incapable of forming cross-linkers, were also separated from the fibroin network and formed keratin clusters. After a certain time, these clusters diffused and positioned themselves among the porous structure of the silk fibroin networks. This explains why the morphology of the naturally self-assembled samples with less than 90 *wt* % silk exhibited both porous and flake-like regions. Therefore, between the two methods, ultrasonication is a more effective way to produce hydrogel composite systems that are stable, homogenous, and well-blended without phase separation.

## 3. Experimental Section

### 3.1. Material Preparation

#### 3.1.1. Silk Solution

The general preparation process of silk aqueous solution has been discussed previously [4]. Raw *B. mori* silkworm cocoons (China) were boiled for 30 min in a 0.02 M Na_2_CO_3_ solution to remove sericins—the glue like protein coated on silk fibers [4]. The remaining silk fibroin fibers were washed in distilled water and air dried in the hood overnight. After drying, the silk fibroin was dissolved in a 9.3 M LiBr solution for 4 h at 60 °C, and then dialyzed in the distilled water for three days. Finally, the solution was centrifuged and filtered, resulting in a 10% (*wt*/*v*) silk fibroin aqueous solution.

#### 3.1.2. Wool Solution

Half a gram of natural sheep wool (Australia) was defatted in a soxhlet solution with approximately 400 mL of hexane for two days. The defatted wool was then dissolved in a mixture solution with 0.728 mL of 8 M urea, 10 mL of NaOH, and 1.73 mL of 0.2 M SDS for 2–3 days at 70 °C. Then, the wool solution was dialyzed against distilled water for three days and centrifuged to collect the supernatant, resulting in 3.3% (*wt*/*v*) wool keratin aqueous solution. Finally, the silk and keratin solutions were both diluted to 3 *wt* % with distilled water and then slowly mixed at different mass ratios (see below) using a pipet to avoid protein aggregation.

#### 3.1.3. Ultrasonication-Induced Hydrogel

A VC750 Ultrasonic Processor (Sonics & Materials, Inc., Newtown, CT, USA), which consists of a power supply of 750 watts and a frequency of 20 kHz, was used to sonicate the silk/keratin blend solutions and induced gelation. Sonication, also known as ultrasonication, is the use of ultrasonic frequencies from an ultrasonic probe to agitate particles in the sample. In this study, ultrasonic waves are used to initiate self-assemble/crystallization processes in the silk fibroin/keratin solutions [5].

Two milliliters eachof silk/keratin aqueous solutions with varied compositions (SKS100, SKS90, SKS70, SKS50, SKS30, SKS10, and SKS0, where SKS stands for “silk/keratin-sonicated” and the number represents the weight percent of silk fibroin in the silk/keratin blend) were loaded in a 5 mL tube and ultrasonicated for 10 s at 30% maximum amplitude. Sol-gel transition or protein aggregation was observed in a short time between 2 min and 20 min for all samples.

#### 3.1.4. Naturally Assembled Hydrogel

Two milliliters each of silk/keratin aqueous solutions with varied compositions (SKN100, SKN90, SKN80, SKN70, SKN50, SKN30, SKN20, and SKN10, where SKN stands for “silk/keratin-natural assembly” and the number represents the weight percent of silk fibroin in the silk/keratin blend) were loaded and stirred in a 5 mL tube. The solutions were sealed and left at room temperature for two days to form hydrogels/aggregates naturally.

Finally, the hydrogels/aggregates generated from both ultrasonication (Method 1) and self-assembled (Method 2) methods were placed prefrozen in liquid nitrogen and immediately lyophilized in −80 °C to avoid structural changes. The lyophilized hydrogels or aggregates were used for various material characterizations and analysis.

### 3.2. Fourier Transform Infrared Spectroscopy (FTIR)

FTIR analysis of silk/keratin hydrogel scaffolds was performed using a FTIR spectrometer (Tensor 27, Bruker Optics Inc., Billerica, MA, USA), equipped with a deuterated triglycine sulfate detector and a multiple reflection, horizontal MIRacle ATR attachment (using a Ge crystal, from Pike Tech, Madison, WI, USA). The instrument was continuously purged withnitrogen gas to eliminate the spectral contributions of atmospheric water vapor. For each measurement, 128 scans were co-added with resolution 4 cm^−1^, with the wavenumbers ranging from 400 to 4000 cm^−1^. Fourier self-deconvolution (FSD) of the IR spectra covering the Amide I region (1595–1705 cm^−1^) was performed using Opus 5.0 software [4]. Deconvolution was performed using Lorentzian line shape with a half-bandwidth of 25 cm^−1^ and a noise reduction factor of 0.3, and the self-deconvoluted spectra were then curve fitted by subsequent Gaussian peaks. FSD is a common signal-processing tool that allows deconvolution of overlapping bands [4]. Using a high pass filter, the broad and indistinct Amide I band (C=O stretching bonds in protein backbones) can be narrowed synthetically to provide a deconvoluted spectrum with better peak resolution. The deconvoluted Amide I spectra were area normalized, and the relative areas of the single bands were used to determine the fraction of the secondary structures in the protein hydrogel scaffolds.

### 3.3. Differential Scanning Calorimetry (DSC)

The freeze-dried silk/keratin hydrogel scaffolds (each about 5 mg) were encapsulated in Al pans and heated in a TA Instruments DSC (Q100, TA Instruments Co., Ltd., New Castle, DE, USA), with purged dry nitrogen gas flow (50 mL·min^−1^), and equipped with a refrigerated cooling system. The instrument was calibrated with indium and sapphire for temperature and heat flow. Standard mode DSC measurements were performed at a heating rate of 2 °C·min^−1^. Temperature-modulated differential scanning calorimetry (TMDSC) measurements were also performed at a heating rate of 2 °C·min^−1^ with a modulation period of 60 s and temperature amplitude of 0.318 °C. Aluminum and sapphire reference standards were used for calibration of the heat capacity. All aluminum sample pans were paired with a same weight.

### 3.4. Scanning Electron Microscopy (SEM)

The surface morphologies of silk/keratin hydrogel scaffold samples were observed with SEM (LEO 1530 VP, Carl Zeiss Microscopy LLC, Thornwood, NY, USA). Samples were broken in liquid nitrogen first and fixed on the SEM holder by conducting tape. Then they were coated with platinum by a 20 s plasma deposition (Desk II, Denton Vacuum Inc., Moorestown, NJ, USA) for the SEM measurements.

### 3.5. Atomic Force Microscope (AFM)

AFM imaging was used to identify the nanoscale morphological features of silk-keratin samples. AFM was performed in tapping mode on a Park Systems (PSIA) XE100 Scanning Probe Microscope (Park Systems Inc., Santa Clara, CA, USA) using rotated tapping-mode etched silicon probes. Both topography and phase signal images were recorded with different sizes in the ambient air, with typical scan rates of 1.5 Hz. Selected silk-keratin solutions or aggregate emulsions (see the Section 2.2. for details) with a diluted concentration of 0.01 *wt* % were dropped on mica surfaces to avoid the impact of substrates.

## 4. Conclusions

Ultrasonication is a quick, effective method of physically attracting keratin filaments with silk fibroin chains into a stable, homogenous composite at the nanoscale. When the silk fibroin concentration is at least 70 *wt* %, the system can continue to form a stable hydrogel network at the microscale with consistent morphology. The mechanism of gelation in this system is due to the formation of intermolecular β-sheet crystals, connecting fibroin-keratin blended chains together. Naturally self-assembled systems, on the other hand, cannot form a stable fibroin-keratin linked structure at the nanoscale. However, high β-sheet crystallinity can still help the system self-assemble to become a stable hydrogel system with partial phase separation. The findings of this research can be beneficial to improve the properties of hydrogel composite biomaterials and to develop other biocompatible medical devices with multiple components in the future.

## Figures and Tables

**Table 1:** Percentage of secondary structures in ultrasonicated silk fibroin/keratin blends (all calculated secondary structure fractions have a same unit (*wt* %) with a ±2 *wt* % error bar).

Sample	Silk Fraction in Sample (%)	β-Sheet Crystallinity (B)	α-Helix & Random Coils (A + R)	Turns (T)	Side Chains (S)
SKS0	0	25.5	44.3	26.5	3.7
SKS10	10	28.3	43.1	24.4	4.2
SKS20	20	30.5	43.8	24.1	1.6
SKS30	30	34.2	41.9	22.3	1.7
SKS50	50	35.3	41.2	21.9	1.6
SKS70	70	43.2	31.5	21.6	4.3
SKS80	80	48.8	27.1	20	4.1
SKS90	90	50.2	22.8	19.2	7.8
SKS100	100	54.5	22.9	19.3	3.2

**Table 2:** Percentage of secondary structures in naturally self-assembled silk fibroin/keratin blends (all calculated secondary structure fractions have a same unit (*wt* %) with a ±2 *wt* % error bar).

Sample	Silk Fraction in Sample (%)	β-Sheet Crystallinity (B)	α-Helix & Random Coils (A + R)	Turns (T)	Side Chains (S)
SKN0	0	22.6	45.7	25.6	6.1
SKN10	10	24.8	44.5	29.1	1.5
SKN30	30	32.4	41.3	24.3	2.1
SKN50	50	41.5	36.3	21.8	0.3
SKN70	70	45.7	32.4	21.4	0.5
SKN90	90	51.1	28.6	19.5	0.7
SKN100	100	52.4	28.5	18.6	0.4

**Table 3:** Thermal properties of ultrasonication-induced silk fibroin/keratin hydrogel blend scaffolds.

Sample	Glass Transition Temperature (°C)	∆*C_p_* (J·g^−1^·°C^−1^)	Degradation Peak (°C)
SKS0	153.5 ^1^	0.159	274.3
SKS10	154.0 ^3^	0.189	293.3
SKS20	155.1 ^7^	0.200	296.3
SKS30	159.7 ^8^	0.202	299.6
SKS50	162.7 ^8^	0.215	276.1
SKS70	164.8 ^0^	0.222	275.1
SKS80	171.0 ^0^	0.243	273.9
SKS100	183.9 ^0^	0.250	273.1

All the superscript are the last reading figures from the software.
